# The Effect of the Combination of Two Postbiotics on Anxiety-like Behavior in Animal Models

**DOI:** 10.3390/cells13232006

**Published:** 2024-12-05

**Authors:** Vanesa Robles, Ferran Balaguer, Miren Maicas, Juan Manuel Martínez-Vázquez, Patricia Martorell, Marta Tortajada, Daniel Ramón, David G. Valcarce

**Affiliations:** 1 Cell Biology Area, Molecular Biology Department, Campus de Vegazana s/n, Universidad de León, 24071 León, Spain; v.robles@unileon.es; 2 Archer Daniels Midland, Nutrition, Health & Wellness, Biopolis S.L. Parc Científic Universitat de València, C/Catedrático Agustín Escardino Benlloch, 9, 46980 Paterna, Spain; ferran.balaguer@adm.com (F.B.); miren.maicas@adm.com (M.M.); marta.tortajada@adm.com (M.T.); daniel.ramonvidal@adm.com (D.R.); 3 Instituto Español de Oceanografía, Centro Oceanográfico de Santander (COST-IEO), CSIC, Calle Severiano Ballesteros 16, 39004 Santander, Spain; jmanuel.martinez@ieo.csic.es; 4 Animal Health and Production, Veterinary Public Health and Food Science and Technology Department Faculty of Veterinary Medicine, University Cardenal Herrera CEU, C/Tirant lo Blanc 7, 46115 Alfara del Patriarca, Spain

**Keywords:** *Bifidobacterium*, *Lacticaseibacillus*, anxiety-like, postbiotics

## Abstract

With increasing evidence showing the connections between the microbiome, neurophysiology, and behavior, our research endeavors to investigate whether the consumption of a combination of two postbiotics with antioxidant effects can affect behavior regulation in model species. Here, we worked with a combination (1:1 ratio) of heat-treated *Bifidobacterium longum* subsp. *longum* ES1 (CECT7347) and *Lacticaseibacillus rhamnosus* BPL15 (CECT8361) as a dietary supplement. To examine the potential benefit of using this formulation to alleviate anxiety-like behavior, we employed two model species, *Caenorhabditis elegans* and adult *Danio rerio*. In *C. elegans*, the postbiotic supplementation reduced the anxiety-related behavior analyzed by means of the octanol avoidance test. In zebrafish, the novel tank test indicated a different swimming pattern 2 and 4 months after the animals were fed with the postbiotic combination. While fish did not exhibit any variance in their locomotion parameters such as pace and speed, they showed a statistically significant preference to spend more time in the upper zone of the water tank, a behavior that is correlated with a lower anxiety-like behavior in these species. Our aim with this study is to present evidence that can be used to develop whole-cell postbiotic-based novel and innovative dietary supplements for anxiety-related conditions.

## 1. Introduction

Anxiety disorders are defined by excessive fear and worry, as well as associated behavioral disturbances, that cause significant distress or impairment in functioning [1]. Nowadays, anxiety disorders are the most prevalent mental disorders and generally start before or in early adulthood [2]. According to the Global Health Data Exchange (GHDx) database [3] in 2019, approximately 301 million individuals were affected by anxiety disorders, including 58 million children and adolescents. Due to the COVID-19 pandemic, the number of individuals affected by anxiety and depressive disorders significantly increased in 2020 [4]. Various types of anxiety disorders exist, such as generalized anxiety disorder, panic disorder, social anxiety disorder, and separation anxiety disorder, with specific major diagnostic features [1].

Pharmacotherapy using selective serotonin/serotonin–noradrenaline reuptake inhibitors (SSRIs/SNRIs) [5,6] (the most prescribed drugs), psychotherapy, or a combination of both are the main approaches to anxiety disorders. However, given the global impact of anxiety disorders and mental illnesses, the scientific and medical community are continuously researching new pharmacological and nonpharmacological treatments addressing these conditions. Currently, there is a growing interest in studying the impact of dietary supplements [7,8], including probiotics [9,10], as complementary tools to support individuals in this demographic cohort. The interest in nutritional psychiatry is based on an increasing body of evidence suggesting the presence of direct connections between nutrition, mental function, mental health, and susceptibility to stress across the lifespan [11].

Probiotics are live microorganisms, which when administered in adequate amounts, confer a health benefit to the host [12]. The use of probiotics as a supplement in mental health disorders is based on compelling clinical and preclinical evidence supporting the interaction between the gut microbiome and the nervous system, which affects both adaptive and dysfunctional neurological processes [13]. This bidirectional communication between the nervous system and the gastrointestinal tract is being currently described as the gut–brain axis [14]. In this context, the gut microbiome influences the development and function of the nervous system through three primary mechanisms, namely the modulation of the immune response, the impact on metabolism, including hormones, neuropeptides, and neurotransmitters, and direct effects on neurons and neuronal signaling [13]. Gut changes due to variations in the gut microbiome can affect host behaviors, including social activity, stress, and anxiety responses, which are associated with neuropsychiatric conditions [15]. Moreover, the intake of ingredients with antioxidant capacity has been associated with improved depression and anxiety states [16].

In the same field of study and during the last decade, the use of postbiotics has gradually gotten the attention of scientific and nutrition-focused communities. Postbiotics were defined in 2021 by the International Scientific Association for Probiotics and Prebiotics (ISAPP) as a “preparation of inanimate microorganisms and/or their components that confer a health benefit on the host” [17]. Postbiotics are an attractive option for new formulations, as inactivated organisms do not pose the same level of challenge as live organisms in terms of formulation [18]. A notable advantage of postbiotics is their inherent stability during industrial processes and storage, increasing their availability to regions without reliable cold chains [17]. From the functional point of view, they have been proven effective in inhibiting food pathogens, improving gut health, modulating immune response, and showing antioxidant effects [19,20,21]. Additionally, although few, there are some examples of postbiotics with beneficial effects on mental health. In rodents, heat-treated probiotics (MCC1848 *L. helveticus*, EC-12 *E. fecalis*, or ADR-159 *L. fermentum* and *L. delbrueckii*) were able to reduce anxiety-like behavior [14], and the administration of *L. gasseri* HT- CP2305 reduced anxiety and improved sleep quality in humans exposed to chronic stress [22,23]. The fact that postbiotics retain many benefits of probiotics and also present advantages regarding safety and stability makes them good candidates for further research and application.

In this work, we hypothesized that the intake of a combination (1:1 ratio) of bioactive postbiotics based on the heat-inactivated *Bifidobacterium longum* subsp. *longum* ES1 (CECT7347; ES1 HT) and *Lacticaseibacillus rhamnosus* BPL15 (CECT8361; BPL15 HT) could have a positive effect on the amelioration of anxiety associated behavior patterns. To validate our hypothesis, we used *Caenorhabditis elegans* and *Danio rerio* as model species to which behavioral tests were applied for studying the anxiety-like state.

The ES1 HT and BPL15 HT are derived from bacterial strains previously reported to possess beneficial probiotic properties. In a previous study, we demonstrated the antioxidant properties of both probiotic strains in a *C. elegans* model, and a supplementation of a combination of these strains in asthenozoospermic human males decreased the DNA fragmentation and ROS levels in sperm [24]. Moreover, we showed that heat-treated ES1 has the ability to safeguard against oxidative stress damage, reduce acute inflammatory response, prevent disruption of the gut-barrier, and inhibit bacterial colonization via pathways related to innate immune function in a similar way to the alive strain [25]. Moreover, this postbiotic has recently shown the capacity to improve symptom severity in adults with diarrhea-predominant irritable bowel syndrome (IBS) including anxiety scorings [26]. Finally, in a previous study conducted by our group, we demonstrated that the prolonged ingestion of live probiotic *Bifidobacterium longum* subsp. *longum* ES1 and *Lacticaseibacillus rhamnosus* BPL15 reduced anxiety-like behavior in a zebrafish model [27].

Based on these previous references, our objective is to test whether the ES1 HT + BPL15 HT postbiotic combination can beneficially affect anxiety-like behaviors to provide evidence for the potential use of the two bacteria-derived postbiotics as complementary supplements in the field of psychiatry.

## 2. Materials and Methods

### 2.1. Ethical Confirmation Statements

Experiments involving zebrafish were approved by the IACUC of the University of León (Spain) under the authorization number ULE-008-2020. Fish were standard manipulated, and assays fully adhered to the European Union Council Guidelines (2010/63/EU). Experiments were performed following Spanish regulations (RD53/2013) for the use of laboratory animals, as well as the 3Rs principles of human animal experimentation.

### 2.2. Caenorhabditis Elegans Subjects and Maintenance

*C. elegans* N2 Bristol (wild type) strain was provided by the *Caenorhabditis* Genetic Center (CGC), University of Minnesota (USA). The nematodes were routinely propagated at 20 °C on Nematode Growth medium (NGM) plates with *Escherichia coli* OP50 strain as a normal diet, as described [28].

### 2.3. Danio Rerio Subjects and Maintenance

Wild-type zebrafish (AB strain) siblings (4 months old at the start of the trial) were used in this experiment. At the start of the experiment, the animals were equally divided based on their weight and housed in 3 L water tanks in groups of 10 animals per tank and per tested condition. All water tanks were provided with a recirculating system with constant water exchange (Aquatic Habitats, Apopka, FL, USA). All fish were raised and maintained in standard conditions [29] (temperature of 26 ± 1 °C and the room on a 14/10 h light/dark cycle) and fed with a commercial zebrafish diet purchased from Zeigler (Gardners, PA, USA).

### 2.4. Experimental Design

The effect of postbiotic supplementation on behavior modulation was assessed with two animal model species, namely *C. elegans* (trial 1) and *D. rerio* (trial 2).

**Figure 1:**
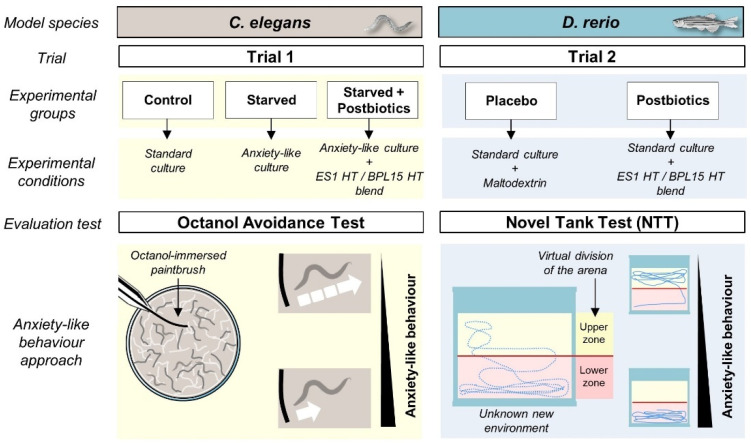
The experimental design including the animal model species used in this project, the experimental groups, the conditions for each trial, and the basis of the chosen evaluation tests for anxiety-like behavior assessment.

Trial 1 included three experimental groups, which were “Control”, “Starved”, and “Starved + Postbiotics” (Figure 1). In all the conditions, young adult stage worms were analyzed. The “Control” group was standard cultured and fed (OP50), as described above. For the anxious-like condition in the “Starved” group, nematodes were incubated in NGM plates without food (OP50) for 20 min. This food deprivation provokes an increase in anxiety due to a decrease in the levels of serotonin [30]. To evaluate the effect of the ES1 HT + BPL15 HT postbiotic combination in the “Starved + Postbiotics” group, nematodes were egg-synchronized in NGM with OP50 plates supplemented with the postbiotic combination (10^8^ cells/plate). At the time of analysis, nematodes were transferred to NGM plates without OP50 in the presence of the postbiotic combination for 30 min. Then, nematodes were transferred and incubated in NGM plates without OP50 for 20 min to apply the anxious condition. Trial 1 was carried out in duplicate with 80 worms per experimental condition.

In trial 2, twenty zebrafish siblings were used. Animals were homogenously divided in terms of weight at the beginning of the experiment into two experimental groups, which were “Placebo” and “Postbiotics” (Figure 1). Each fish group had different feeding regimes, divided in two daily dosages; the control “Placebo” group received daily the commercial diet and 0.22 g maltodextrin, while the “Postbiotics” group received the commercial diet and the ES1 HT + BPL15 HT postbiotic combination (1:1 ratio, 10^9^ CFU in one capsule containing 0.22 g maltodextrin). The technical personnel in charge of the maintenance, feeding, and animal welfare were unaware of the contents of the capsules and the animals were equally manipulated in routine procedures. The experimental groups were blindly numbered. Fish were tested for their behavioral patterns 2 and 4 months after the beginning of the trial.

### 2.5. Postbiotic Combination Preparation and Administration

In trial 1, *Bifidobacterium longum* ES1 (CECT7347) and *Lacticaseibacillus rhamnosus* BPL15 (CECT8361) bacterial cultures were grown in Man–Rogosa–Sharpe (MRS) medium agar and broth supplemented with 0.05% (*w*/*v*) cysteine and kept at 37 °C in anaerobic conditions for 24 h. For heat inactivation, cells were harvested by centrifugation, washed twice with saline solution, and autoclaved at 121 °C, 1 bar, for 20 min. In the “Starved + Postbiotics” group in *C. elegans*, the nematodes were egg-synchronized in NGM with OP50 plates supplemented with the ES1 HT + BPL15 HT postbiotic combination (1:1 ratio, 10^8^ cells/plate).

In trial 2, 0.22 g maltodextrin (placebo) and postbiotic blend (1:1 ratio, 10^9^ CFU in one capsule containing 0.22 g maltodextrin) were used as supplements and were provided as capsules by ADM Biopolis. The content of each capsule was split into two and each dose (half of the capsule content) was provided to the experimental fish in the rearing water of the housing tanks 30 min prior to each routine feeding (twice a day) as a measure to guarantee postbiotic blend ingestion. These feeding conditions were maintained throughout the entire experiment (4 months). The fish were fed twice a day, seven days/week, at 08:30 h and 13:30 h.

### 2.6. Nematode Behavioral Assay

An avoidance assay was used with the nematode model as an anxiety-related behavior. The experiments were carried out using the “smell-on-a-stick” assay previously described [30]. Briefly, the blunt end of a hair from a Loew-Cornell (Loew-Cornell, Teaneck, NJ, USA) 9000 Kolinsky 7 paintbrush, taped to a Pasteur pipette, was immersed in freshly prepared 30% octanol in EtOH (vol/vol) and placed in front of a forward-moving nematode. The amount of time the worm took to begin backward movement (octanol avoidance) was measured using a laboratory timer.

### 2.7. Zebrafish Behavioral Assay

To test the exploratory behavior of the animals, we used a novel tank test (NTT). The protocol was carried out as follows: each animal was carefully withdrawn from its housing tank and placed at the bottom of an unknown quadrangular glass aquarium (20 cm × 8 cm × 18 cm; length × width × depth; swimming volume: 3.5 L). The fish were filmed (1920 × 1080 px) for 6 min. The first minute was considered as the acclimation time to the new environment, and the behavior analysis (the actual NTT) was considered for the remaining 5 min. Individual swimming activity was analyzed using Noldus Ethovision^®^ tracking software (v. XT16, Wageningen, the Netherlands), generating a virtual grid dividing the tank in 2 zones (upper and lower; Figure 1). A metrical reference was included in the recorded frame for posterior evaluation of the kinetic parameters (velocity and total distance swam) of each animal. Zone preference in terms of time spent in each zone and latency to the first entry in the upper zone were used as variables to evaluate the swimming pattern.

### 2.8. Zebrafish Biometrical Analysis

The growth evaluation was performed at 2 and 4 months after behavioral post diet supplementation. Animals were individually anesthetized just after the end of the NTT by immersion in a 110 mg/L buffered tricaine methane sulfonate (MS222), (Sigma-Aldrich, Steinheim, Germany) solution. Approximately 30 s after immersion, when gill movements were evidently reduced, fish were weighed using a microbalance (Mettler MT5, Mettler Toledo, Cornellà de Llobregat, Spain) to an accuracy of mg. The fish were afterwards photographed under a stereomicroscope (Nikon SMZ-U; Nikon, Japan) and subsequently placed in a recovery tank with a freshwater system. Photographs were processed with Adobe Photoshop CC 2018 software (San José, CA, USA), establishing a conversion between pixel size and units of length (accuracy of 0.01 mm).

### 2.9. Statistical Analysis

The GraphPad Prism 9.0.0 package (GraphPad Software, Inc., San Diego, CA, USA) was used for statistical analysis and figure generation. A Shapiro–Wilk test was run for all variables to check normality. In the *C. elegans* trial, an octanol avoidance assay was analyzed using the non-parametric Kruskal–Wallis test. In the *D. rerio* experiment, the differences between experimental groups were identified using Student’s t test for normally distributed variables. For non-parametric data, a Mann–Whitney test was run. All data are shown as mean ± SEM (* *p* < 0.0500; ** *p* < 0.0100; *** *p* < 0.0010; **** *p* < 0.0001; ns: no significant changes).

## 3. Results

### 3.1. Postbiotic Supplementation Reduces the Time to Response to an Avoidance Stimulus Under Anxiety-like Conditions in C. elegans

**Figure 2:**
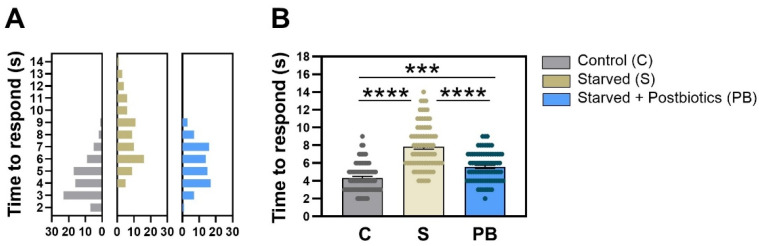
The modulation of the time of response in avoidance of octanol in *C. elegans*. (**A**) The frequency of distribution of the times recorded for each nematode in each of the groups (bin size = 1). (**B**) Mean values of the response time in each experimental group. “Control” refers to nematodes grown under standard conditions; “Starved” refers to worms subjected to starvation (anxiety-like condition); and “Starved + Postbiotics” refers to individuals supplemented with the ES1 HT + BPL15 HT postbiotic blend and subjected to starvation (anxiety-like condition). Asterisks show significant differences (**** *p* < 0.0001; *** *p* < 0.0010).

Figure 2 shows the response of *C. elegans* to an avoidance stimulus (octanol) in the three experimental groups in terms of the time it took for the nematodes to start the backward movement when they noticed alcohol presence. The frequency distribution of the response time values recorded for each nematode in each group in trial 1 is represented in Figure 2A. Histograms show a clear variation in the distribution of the variable among treatments. The condition without food (“Starved” group), stimulating the anxious-like behavior, had a delayed reaction time to avoid octanol than nematodes fed with *E. coli* OP50 strain (*p* < 0.0001; Figure 2B). Nematodes treated with the ES1 HT + BPL15 HT postbiotic combination significantly reduced the avoidance time when worms were exposed to anxious conditions (*p* < 0.0001; Figure 2B), although statistical differences were also found between the “Control” group and the anxiety-induced nematodes fed with postbiotics, “Starved + Postbiotics”, suggesting that the postbiotics did not completely counteract the anxiety-like behavior (*p* < 0.0010; Figure 2B). Altogether, the ES1 HT + BPL15 HT postbiotic combination was able to reduce the induced anxiety-related behavior.

### 3.2. Postbiotic Supplementation in the Diet Does Not Affect Weight or Length in Zebrafish

**Figure 3:**
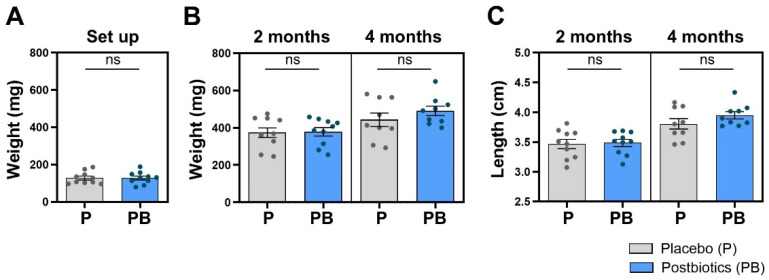
Biometrical mean values. (**A**) At the beginning of the experiment, experimental groups were homogenously set up in terms of fish weight. (**B**) Weight mean values at 2 and 4 months of experimentation. (**C**) Length mean values at 2 and 4 months of experimentation. “Control” refers to zebrafish diet-supplemented with maltodextrin and “Postbiotics” refers to zebrafish diet-supplemented with the ES1 HT + BPL15 HT postbiotic blend (ns—no significant changes). Dots represent values from individual animals within each experimental group.

The initial homogeneity in the weight of the zebrafish included in trial 2 before the start of the experiment (Figure 3A) was maintained in the two subsequent samplings (Figure 3B). No significant differences were found between the “Placebo” and “Postbiotics” groups neither at 2 months (*p* = 0.4439) nor at 4 months post diet supplementation (*p* = 0.1503). The length values followed the same pattern, with no significant differences in the 2-month (*p* = 0.4280) or 4-month sampling (*p* = 0.0995) (Figure 3C).

### 3.3. Kinetic Parameters Are Not Affected by Postbiotic Ingestion in the Zebrafish Model

**Figure 4:**
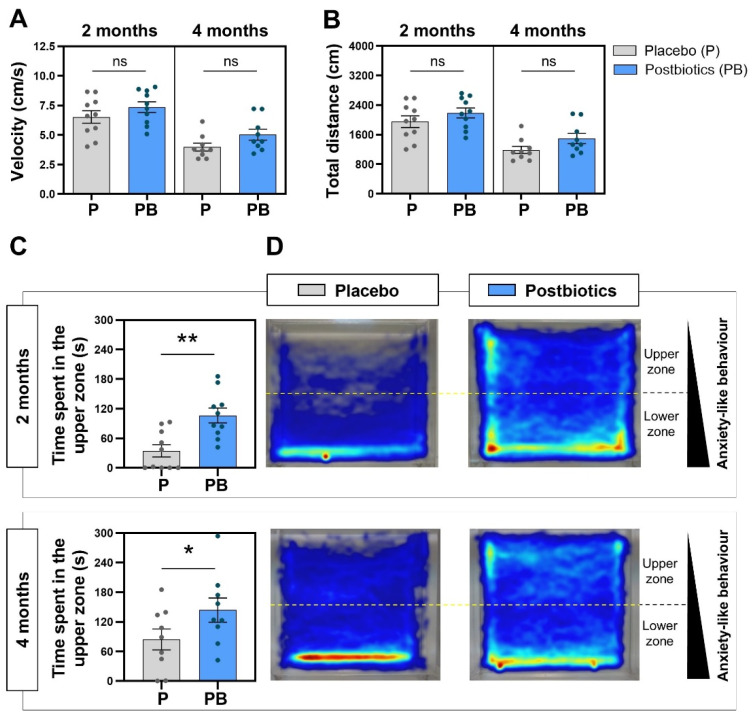
Analysis of the zebrafish swimming pattern at 2 and 4 months. (**A**) Fish velocity during the NTT. (**B**) Total distance swam by the fish during the NTT. (**C**) Time spent in the upper zone of the novel tank during the NTT. (**D**) Merge heatmaps of all the trajectories of each experimental group. “Control” refers to zebrafish diet-supplemented with maltodextrin and “Postbiotics” refers to zebrafish diet-supplemented with the ES1 HT + BPL15 HT postbiotic combination. (* *p* < 0.0500; ** *p* < 0.0100; ns—no significant changes). Dots represent values from individual animals within each experimental group.

Globally, the kinetics of the studied fish showed similar results between groups (Figure 4A,B), reporting no statistically significant differences at any evaluation timepoint. Interestingly, both studied parameters (velocity and total distance swam) showed higher mean values at the 2-month evaluation timepoint compared to the 4-month evaluation timepoint, and the maximum velocity and distance values corresponded to the fish included in the “Postbiotics” group and were evaluated at both timepoints.

### 3.4. Postbiotic Ingestion Modulates the Exploration Pattern in the Zebrafish Model

The postbiotic blend tested in the zebrafish model indicated the ability of zebrafish to modulate the zone chosen to spend more time in. Data regarding the exploration pattern in each experimental group are included in Figure 4C and 4D. At 2 months post diet supplementation, the postbiotic-supplemented group spent 106.2 ± 14.98 s in the upper zone compared to the 34.64 ± 12.48 s for the placebo group (Figure 4C), with statistically significant differences between two groups (*p* = 0.0024). At 4 months post diet supplementation, the fish showed a different swimming pattern during the NTT, with statistically significant differences between groups (*p* = 0.0423; Figure 4C). The heatmaps generated by the Noldus Ethovision^®^ tracking software allow us to observe in a simple qualitative way the behavioral differences in the two experimental groups (Figure 4D). The different exploration pattern is evident, finding that the fish from the “Placebo” group remain for a longer time in the lower zone of the novel tank compared to their counterparts from the “Postbiotic “group.

**Figure 5:**
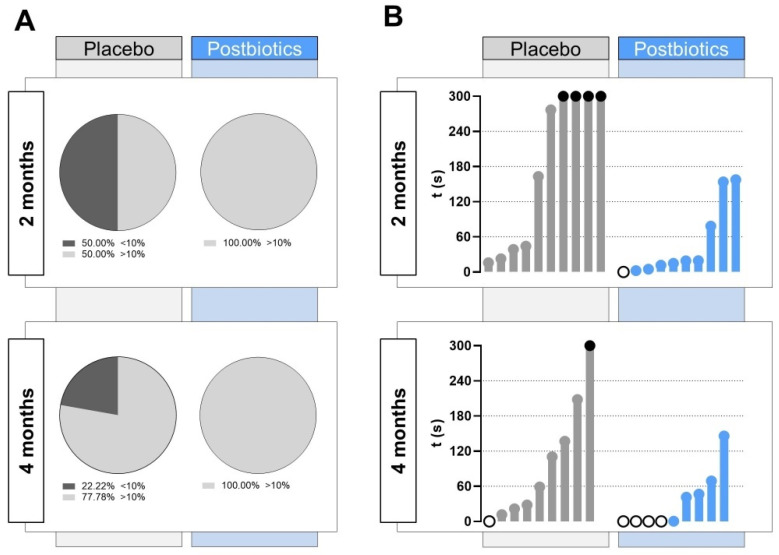
Detailed study on the zebrafish swimming pattern in the upper zone of the tank. (**A**) Pie chart representing the percentage of fish spending less than 30 s (10% of the total NTT; stripped black) in the upper zone during the behavior assay at 2 months and 4 months after the beginning of the experiment in each experimental group. (**B**) Latency to the first entry in the upper zone. “Placebo” refers to zebrafish diet-supplemented with maltodextrin and “Postbiotics” refers to zebrafish diet-supplemented with the ES1 HT + BPL15 HT postbiotic combination. White dots represent fish swimming in the upper zone at the beginning of the NTT and black dots represent fish swimming throughout the NTT (300 s) in the lower zone.

Furthermore, a deeper study on the zebrafish swimming pattern in the upper zone of the tank was performed, establishing a threshold of 30 s (10%) of the total NTT (Figure 5A). At 2 months post diet supplementation, half of the animals included in the “Placebo” group did not reach the established threshold, whereas all the individuals in the “Postbiotics” group spent in total more than 30 s in the upper zone of the novel tank. At 4 months post diet supplementation, some of the fish in the “Placebo” group did not score over 30 s in the upper zone (22.22%), whereas all fish exceeded the threshold of 30 s in the “Postbiotics” group.

After 1 min of acclimatization, the latency to the first entry in the upper zone shown by the animals in each experimental group was quantified. As can be seen in Figure 5B, at 2 months post diet supplementation, four fish in the “Placebo” group did not score time in the upper zone of the tank, and the latency to rise was delayed above 2 min for two of them. In the “Postbiotics” group, the latency values were globally lower, with an individual swimming already in the upper zone at the beginning of the NTT. After 4 months post diet supplementation, the trend softened in the “Placebo” group, although it continued to show lower values in “Postbiotics” group, in which up to four fish were already in the upper zone of the tank at the beginning of the recording after the acclimatization minute.

## 4. Discussion

In a previous report, a reduced anxiety-like behavior in the zebrafish model was shown with the prolonged ingestion of a combination of the probiotics Bifidobacterium longum subsp. longum ES1 (CECT7347) and *Lactocaseibacillus rhamnosus* BPL15 (CECT8361) [27]. Our findings support the extensively reported evidence in animal and human studies [31,32] suggesting that probiotics exhibit promising outcomes in connection with mental health pathways. In the current study, we wanted to explore whether the combination of the two postbiotics generated by heat treatment of their respective probiotic strains could be used as supplements with mental health benefits. The desirable characteristics of postbiotics, such as their safety, resistance to hydrolysis, non-toxicity, stability under digestive conditions, and longer shelf life, make them attractive options [33]. To date, experimental approaches aiming to use postbiotic formulations to reduce anxiety-linked parameters are scarce, although some experiments have been performed. It was demonstrated that the administration of a heat-inactivated *Lactobacillus gasseri* strain to a group of students under a stressful event slowed down the increase in salivary cortisol as compared to the placebo group [22]. Other experiments performed with the same postbiotic showed a reduction in anxiety test scores in individuals with postbiotics supplemented in their diet compared to the placebo cohort [23]. Moreover, the administration of *Lacticaseibacillus paracasei* PS23 in rodents showed that both alive and heat-treated cells could reverse chronic corticosterone-induced anxiety [34]. To assess our goal, we have performed two behavioral trials using two model organisms, namely *C. elegans* (trial 1) and *D. rerio* (trial 2). These two model species are extensively used for high-throughput chemical screening and predictive toxicology [35,36]. Furthermore, the two models share numerous biological processes with rodents and humans, along with multiple genes and neuronal pathways that are highly conserved throughout evolution. *C. elegans* is extensively employed as a model organism for a diverse range of scientific research [37]. In our group, this model has allowed us to identify new bacterial strains with antioxidant properties as probiotics [38,39] or postbiotics [25]. Moreover, *C. elegans* is considered a valuable organism for behavioral experiments, since it has a relative complex neuronal network that consists of 302 neurons with a great cellular diversity, including the main types of neurons present in mammals such as dopaminergic, cholinergic, or serotonergic neurons that exert a strong impact on physiology and behavior [40]. In the current work, we have used the *C. elegans* octanol avoidance test as a tool for the evaluation of anxiety [30]. To assess the potential modulation effect of our ES1 HT + BPL15 HT postbiotic combination on anxiety-like-behavior, anxiety was induced by starvation of the nematodes divided into two groups (“Postbiotic”-supplemented group and “Placebo” group). Sensitivity to octanol decreased when animals were off food or when serotonin levels decreased [30]. We evaluated the amount of time it took for the animal to initiate backward movement when exposed to the avoidance stimulus. Our findings clearly demonstrated a modulation of the behavioral response in worms supplemented with the postbiotic combination (Figure 2). The animals exposed to anxiety-inducing conditions exhibited a poor response to octanol, resulting in a significant delay in their response time compared to the control group. On the contrary, the group that received postbiotic supplementation and was subjected to anxiety-inducing conditions responded similarly to the control group. The promising findings of this initial *C. elegans* behavioral trial provided us a rationale to further validate these results in a vertebrate model such as zebrafish.

Numerous experimental procedures have been employed to examine zebrafish behavior, often adopting approaches commonly used in mouse models. For instance, classical tests like choice [41], place preference [42], or light/dark preference [43] tests have proven effective in investigating the neural mechanisms of social behavior, exploring fear and anxiety, and assessing the impact of psychoactive substances and genetic alterations [44,45,46,47]. In the present work, we use the novel tank test (NTT) to validate our hypothesis. The NTT is a commonly employed technique in zebrafish behavior experiments, which evaluates the response of zebrafish to a new unknown environment [48,49,50,51,52]. The NTT can be regarded as an analogous counterpart of the rodent open-field paradigm, as it examines the zebrafish’s geotaxis escape diving instinct in specific experimental conditions, with a conflict between “safe” diving behavior and “exploration” swimming behavior. In normal laboratory settings, the zebrafish typically tend to spend more time at the bottom of the tank, showing erratic movements and immobility events during the initial minutes of monitoring [53]. Following the habituation period, the zebrafish gradually explore the upper regions of the water column. The premise underlying this assumption is that when fish are introduced to an unfamiliar environment, their natural instincts prompt them to exhibit a defensive response by diving to the bottom of the water column, thereby minimizing their chances of being preyed upon by a predator near the surface [54]. In our zebrafish trial, the zebrafish supplemented with postbiotics exhibited a notable decrease in their geotactic behavior towards the bottom of a new tank (Figure 4), which could be associated with a lower level of anxiety. The reduction in the time spent by the zebrafish in the lower zone of the tank is a behavioral pattern commonly found in studies focused on analyzing the effect of anxiolytic compounds. For example, zebrafish exposed to the psychoactive metabolite noribogaine (potentially useful to treat dependence) showed an increasing percentage of the time spent in the top half zone of the tank [55]. Similar results were found in experiments studying the effects of monoamine oxidase inhibitors on zebrafish behavior [56]. Conversely, studies focused on anxiogenic compounds found opposite results. For example, the combination of the psychostimulant methylphenidate (MPH) and high doses of caffeine induced a strong preferential swimming pattern in the lower zone of the tank [57]. The absence of alterations in kinetic parameters (total distance swam and velocity) in our experiment makes our swimming pattern results even more interesting (Figure 4). The zebrafish moved at an equivalent pace and distance, but they exhibited a tendency to select a specific area of the tank in which they spend more time. Our findings align with prior studies examining the effects of compounds or supplements on zebrafish behavior, in which no alterations in the animals’ locomotion parameters were observed but changes in their preference for a zone were registered [27,55,58]. Another interesting indicator of the potential anxiety-like-behavior reducing capacity of the ES1 HT + BPL15 HT postbiotic combination is the percentage of zebrafish that spent most of their time in the upper zone of the tank (Figure 5). While in the “Placebo” group, we recorded fish not passing the 30 s threshold in the upper area of the tank, in the “Postbiotics” group, the total number of fish exceeded that time in the potentially unsafe zone both at the 2-month and 4-month evaluation timepoints. Similarly, the latency to the first entry into the upper zone of the tank exhibited a distinct pattern (Figure 5) between two groups. In the “Placebo” group, some fish did not enter the upper zone during the entire NTT, a finding that was not observed in the “Postbiotic” group, where more fish were observed to be swimming in the upper zone immediately after the acclimatization period. This behavioral pattern was also observed in our previous investigation involving the use of the probiotic *Lactocaseibacillus rhamnosus* CECT8361 and *Bifidobacterium longum* subsp. *longum* CECT7347 strains [27], which were used to create the ES1 HT + BPL15 HT postbiotic combination tested in this study.

## 5. Conclusions

The ingestion of a combination of two postbiotics generated from two heat-treated bacteria (*Bifidobacterium longum* subsp. *longum* ES1 and *Lactocaseibacillus rhamnosus* BPL15), previously identified as probiotic bacteria, with antioxidant activity can decrease anxiety-like responses in two model species, namely *C. elegans* and *D. rerio*. We used behavior test analysis to reveal distinct patterns in the groups supplemented with the two postbiotic combinations. These basic research results provide initial evidence which may lead to new potential strategies for using the two tested postbiotics in modulating anxiety-related behaviors that should be further evaluated in clinical trials with human subjects.

## 6. Patents

The patent PCT3113.19 results from the work reported in this manuscript.

## Data Availability

Data is contained within the article.
